# Coronavirus Disease 2019 (COVID-19)-Induced Hyponatremia: A Challenging Geriatric Case

**DOI:** 10.7759/cureus.72202

**Published:** 2024-10-23

**Authors:** Amogh Kiran, Vansh Marwaha, Daravie Pel, Nehal Patel

**Affiliations:** 1 Primary Care, Avalon University School of Medicine, Willemstad, CUW; 2 Surgery, Avalon University School of Medicine, Willemstad, CUW; 3 Primary Care, Saint James School of Medicine, Park Ridge, USA; 4 Family Medicine, Beckley Appalachian Regional Healthcare (ARH) Hospital, Beckley, USA

**Keywords:** covid-19, electrolyte imbalance, geriatrics, hyponatremia, hypoxia, siadh, transaminitis

## Abstract

Coronavirus disease 2019 (COVID-19) has presented numerous challenges to the medical community, including atypical complications such as electrolyte imbalances and organ dysfunction. This case report describes a 91-year-old female with a history of chronic medical conditions, including chronic kidney disease (CKD) stage 3, who presented with hypoxemic respiratory failure, transaminitis, and persistent hyponatremia during the course of her COVID-19 infection. Despite initial suspicion of the syndrome of inappropriate antidiuretic hormone secretion (SIADH), caused by elevated antidiuretic hormone (ADH), the patient's normal osmolality and sodium excretion suggested other contributing factors, such as poor oral intake (per os, PO), volume depletion, and pseudo-acute kidney injury (pseudo-AKI) leading to hypovolemic hyponatremia. Her transaminitis resolved with supportive care, allowing for the safe initiation of remdesivir. The patient's gradual correction of hyponatremia without complications highlights the importance of individualized management. This case highlights the varied and systemic effects of COVID-19, emphasizing the need for comprehensive care and monitoring of electrolyte disturbances during hospitalization.

## Introduction

The most recent public medical issue that has taken its toll on a global scale was the coronavirus disease 2019 (COVID-19) and the resulting pandemic. The virus' prevalence, as well as incident cases, continues to demonstrate itself in clinics and hospitals with varying presentations from patient to patient. One clinical sign that has previously been researched is COVID-19-induced hyponatremia [1]. In particular, hyponatremia is seen in several studies as a laboratory finding that predicts poor prognosis in COVID-19 patients [1,2]. The balance of sodium (as well as other electrolytes) and water is highly regulated in order to maintain the homeostasis necessary for the human body to function normally at a cellular level. The normal plasma tonicity remains at levels of 275-295 mOsm/kg and urine osmolality remains below 100 mOsmol/kg [3]. Hyponatremia, defined as a serum sodium concentration of less than 135 mEq/L, can occur due to a variety of causes, most commonly an abnormality in water content, such as the syndrome of inappropriate antidiuretic hormone secretion (SIADH) [4]. SIADH, when in isolation and in non-association in COVID-19, presents as hyponatremia due to a medical or idiopathic cause that raises antidiuretic hormone (ADH) in the body. Examples of the most common causes of SIADH include the following: central nervous system (CNS) abnormalities that affect the pituitary gland (where ADH is released), cancer (small cell lung cancer), medications (carbamazepine, selective serotonin reuptake inhibitors (SSRIs), cyclophosphamide), surgery, HIV, hormone abnormalities, viruses, and more [3].

COVID-19 predominantly presents as a pulmonary issue, presenting as pneumonia and respiratory symptomatology. Although the pathophysiology of COVID-19's cause of SIADH and hyponatremia is still currently being studied in patients, previous studies have noted several factors, including decreased intravascular volume and decreased extracellular fluid osmolality, stressors of infection, and increased cytokine and inflammatory markers [5]. Previous studies of COVID-19-induced hyponatremia have also demonstrated a higher mortality rate and could be used as a biomarker of prognosis in COVID-19 patients [2]. Our study aims to highlight COVID-19-induced hyponatremia in the following patient case.

## Case presentation

A 91-year-old female with a past medical history of chronic, well-controlled arterial hypertension, type 2 diabetes mellitus, chronic kidney disease (CKD) stage 3, paroxysmal atrial fibrillation, gastroesophageal reflux disease, and major depressive disorder presented to the emergency department with a temperature of 38.6°C (101.5°F), respiratory rate of 26/min with an oxygen saturation of 85%, blood pressure of 130/80 mm Hg, and pulse rate of 100 beats per minute. The patient also reported rhinorrhea, shortness of breath, and non-productive cough, along with sore throat, loss of appetite, asthenia, and headache for the past two days. She denied any recent travel, falls, or sick contacts, and the rest of the review of systems was unremarkable. The patient lives with her husband and is independent in activities of daily living. 

On physical examination, she was found to have coarse breath sounds, dyspnea, dry mucous membranes, oropharyngeal erythema, and mild epigastric tenderness on palpation. The rest of the physical examination was unremarkable.

Initial laboratory workup revealed the following abnormalities as shown in Table 1.

**Table 1:** Laboratory parameters on admission Na⁺: sodium; WBC: white blood cell; AST: aspartate aminotransferase; ALT: alanine aminotransferase; COVID-19: coronavirus disease 2019

Laboratory parameter	Admission value	Normal reference range
Creatinine	1.15 mg/dL	0.6-1.2 mg/dL
Na⁺	131 mEq/L	135-145 mEq/L
WBC count	3.75 x 10⁹/L	4-11 x 10⁹/L
AST	608 U/L	10-40 U/L
ALT	592 U/L	7-56 U/L
COVID-19 viral swab	Positive	Negative
Serum osmolality	285 mOsm/kg	275-295 mOsm/kg
Urinary Na+ excretion	20 mEq/L	20-40 mEq/L
Random serum glucose	206 mg/dL	<200 mg/dL

Based on the clinical and physical examination findings, the patient was found to have hypoxemic respiratory failure and was placed on a 3L nasal cannula for oxygen support.

The patient was treated with nebulized albuterol sulfate (4 mg every eight hours) and IV dexamethasone (6 mg daily for 10 days). Due to transaminitis, remdesivir was initially withheld. On day 4 of admission, the liver enzyme levels normalized, allowing for the initiation of remdesivir.

Despite improvement in her respiratory symptoms, the patient continued to have persistent hyponatremia (sodium (Na⁺): 125 mEq/L at nadir), requiring extended management that included restoring intravascular volume with isotonic saline. 

The patient's sodium levels over the course of hospitalization and follow-up are summarized in Table 2 below.

**Table 2:** Sodium values over the hospital course

Day of hospitalization/follow-up	Sodium level (mEq/L)
Day 1 (admission)	131
Day 4	125 (nadir)
Day of discharge	131
Follow-up (clinic visit)	134

The patient's clinical symptoms and hyponatremia were resolved prior to her discharge. The patient recovered fully from her COVID-19 infection and associated complications. 

## Discussion

The COVID-19 virus, which is known to primarily cause severe hypoxemic respiratory failure, has created a recent challenge for physicians across the world with over 1.2 million deaths and over 104 million cases in the United States alone since its appearance in 2019 [6]. Symptoms may vary including fever, dyspnea/hypoxemia, cough, sore throat, nausea/vomiting, diarrhea, fatigue, headache, and myalgia [7]. There has also been documented evidence of post-acute complications as follows: restrictive pulmonary disease, thromboembolic events, palpitations, myocardial fibrosis, mood disorders, acute kidney injury (AKI), and worsening of underlying endocrine disorders [8]. 

Over the last four years, research has greatly expanded our understanding of the COVID-19 pandemic; however, there is still much to be learned as we continue to combat the repeated waves of COVID-19 variants. While our patient exhibited well-documented signs of COVID-19 such as acute hypoxic respiratory failure, nausea, fatigue, headache, and poor appetite, she additionally presented with acute transaminitis and hyponatremia during her hospital course. 

Given our patient's unremarkable hepatic imaging and self-resolving course, it was possibly secondary to her infection course. Transaminitis has been documented in COVID-19 infection in a study which showed its prevalence in 26% of patients admitted, of whom 31% had recent normal transaminase values. Furthermore, 33% of the patients presenting with transaminitis also required ICU-level care; however, there was no significant association between COVID-related transaminitis on admission and death [9]. Our patient's transient transaminitis delayed the initiation of remdesivir which potentially prolonged her recovery. 

Lastly, another study (Frontera et al.) found hyponatremia was prevalent in one-third of patients infected with COVID-19 and was a significant reason for morbidity and prolonged mechanical ventilation especially due to encephalopathy in hospitalized patients [10]. In addition, the mortality rate was found to be significantly elevated at 23.7% in a recent meta-analysis [1]. The most likely proposed mechanism of hyponatremia is SIADH [10]. However, given that our patient did not have increased sodium excretion, SIADH can be ruled out. The patient's transaminitis, hyperglycemia, and dehydration may have masked the drop in serum osmolality which we would expect in hyponatremia. Thus, her hyponatremia may have been triggered by other factors within the illness course such as poor oral intake versus SIADH.

## Conclusions

COVID-19 continues to challenge healthcare professionals with its varied presentations, ranging from respiratory complications to electrolyte imbalances such as hyponatremia. While SIADH has been explained as the most common cause of hyponatremia in patients infected with COVID-19, other causes such as poor oral intake and solute loss could potentially contribute to its pathogenesis. The patient's transaminitis, another frequent but often overlooked complication of COVID-19, further complicated her management, delaying the use of antiviral treatment. 

This case highlights the importance of individualized patient care in the setting of COVID-19, as each patient may present with unique clinical and laboratory abnormalities. Close monitoring of electrolyte disturbances, prompt management of respiratory failure, and an understanding of the varied systemic effects of COVID-19 are crucial for improving patient outcomes.
